# Implantation einer „small-aperture Intraokularlinse (IOL)“ bei 2 Patientinnen mit irregulärem Astigmatismus nach keratorefraktiver Chirurgie

**DOI:** 10.1007/s00347-020-01274-4

**Published:** 2020-12-04

**Authors:** M. Hartmann, L. Hamon, E. Flockerzi, N. Ardjomand, B. Seitz, L. Daas

**Affiliations:** 1 Klinik für Augenheilkunde, Universitätsklinikum des Saarlandes (UKS), Kirrbergerstr.100, 66421 Homburg/Saar, Deutschland; 2 Sehzentrum für Augenlaser und Augenchirurgie, Graz, Österreich

In unserer Sprechstunde für refraktive Chirurgie stellten sich 2 Patientinnen mit irregulärem Astigmatismus nach keratorefraktivem Eingriff sowie mit vorliegender Cataracta provecta vor. Beide klagten über eine schleichende beidseitige Sehverschlechterung, die mit einer Brille nicht vollständig korrigiert werden konnte. Patientin 1 war 65 Jahre alt und bekam 1990 bilaterale radiäre Keratotomien (RK) in Russland. Patientin 2 war 60 Jahre alt und bekam 2008 extern eine hyperope photorefraktive Keratektomie (PRK).

Die Kataraktoperation bei Patienten nach einem keratorefraktiven Eingriff stellt eine Herausforderung dar. Die Berechnung der IOL wird durch Hornhautaberrationen und das veränderte Verhältnis von Vorder- und Rückflächenkrümmung erschwert [8]. Die IC‑8 (AcuFocus Inc, Irvine, CA, USA) IOL benutzt das Lochblendenkonzept kombiniert mit einer erweiterten Tiefenschärfe und ist ein alternativer Ansatz für die Behandlung von Patienten nach keratorefraktivem Eingriff. Bei irregulärem Hornhautastigmatismus stellt sie eine gute Behandlungsoption dar [5].

Zweck dieser Kasuistik ist es, die Verwendung einer IC-8 (AcuFocus Inc, Irvine, CA, USA) IOL bei 2 Patientinnen mit irregulärem Astigmatismus nach einem vorausgegangenen keratorefraktiven Eingriff zu beschreiben.

## Befunde

**Figure 1:**
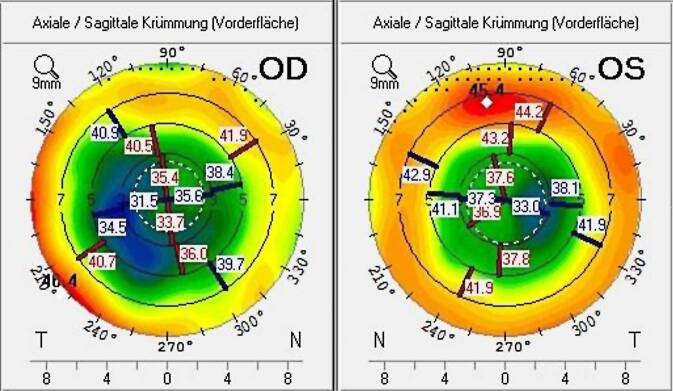

Präoperativ war bei Patientin 1 der bestkorrigierte Fernvisus (subjektive Refraktion rechts +5,75/−1,00/A 55° und links +4,25/−0,50/A 176°) 0,3 LogMAR beidseits. Klinisch waren im Bereich der RK bilaterale stromale Hornhautnarben zu erkennen. Das Hornhautzentrum war aber noch klar, und es bestand eine Cataracta corticonuclearis provecta beidseits. In der Hornhauttopometrie(Pentacam, Oculus Optikgeräte GmbH, Wetzlar, Deutschland)-Analyse zeigte sich links ein irregulärer Astigmatismus, wobei die Hornhaut des rechten Auges deutlich weniger irregulär war (Abb. 1).

**Figure 2:**
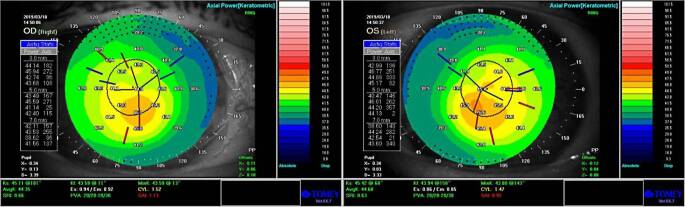

Bei Patientin 2 war präoperativ der bestkorrigierte Fernvisus 0,2 LogMAR rechts und 0,3 LogMAR links (subjektive Refraktion rechts +3,00/−0,75/A 9° und links +3,25/−1,00/A 151°). Klinisch zeigte sich ein regelrechter ophthalmologischer Befund bis auf die beidseitige Cataracta provecta. Es zeigte sich in der Hornhauttomographie(TMS-5, Tomey GmbH, Deutschland)-Analyse links mehr als rechts eine dezentrierte Ablationszone (Abb. 2).

## Therapie und Verlauf

Eine Standardkataraktoperation (Phakoemulsifikation und Implantation einer Hinterkammerlinse) wurde bei beiden Patientinnen durchgeführt, rechts vor links in einem Zeitraum von 3 Wochen bei Patientin 1 und links vor rechts in einem Zeitraum von 5 Tagen bei Patientin 2. Beide Patientinnen wurden postoperativ für 2 Wochen mit einem Kombinationspräparat von Tobramycin 3 mg/ml und Dexamethason 1 mg/ml 5‑mal/Tag sowie hyaluronsäurehaltigen Tränenersatzmitteln versorgt. Der postoperative Verlauf war komplikationslos in beiden Fällen. Für die IOL-Berechnung mit der Haigis-Formel wurde die „totale zentrale corneale Brechkraft“ aus der Pentacam (Oculus Optikgeräte GmbH, Wetzlar, Deutschland) übernommen.

**Figure 3:**
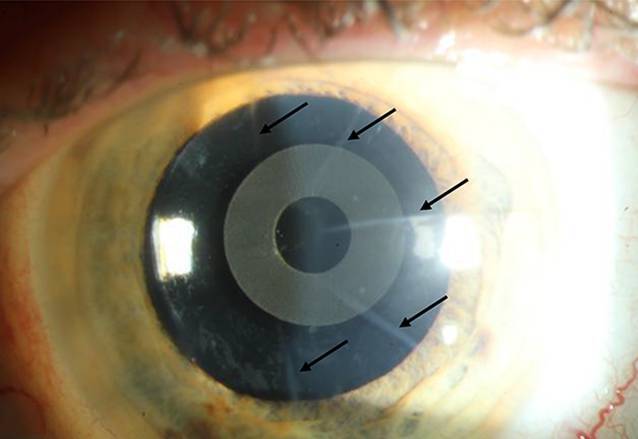

Bei Patientin 1 wurde eine asphärische monofokale Linse (HOYA Vivinex XY1, Surgical Optics, Singapore) +29,0 dpt in das rechte dominante Auge implantiert mit einer Zielrefraktion von −1,0 dpt und die IC-8-IOL (AcuFocus Inc, Irvine, CA, USA) +27,5 dpt in das nichtdominante linke Auge mit einer Zielrefraktion von −0,50 dpt. Der unkorrigierte postoperative Fernvisus nach 6 Monaten betrug 0,1 LogMAR monokular beidseits und 0,0 LogMAR binokular, und der binokulare Nahvisus lag bei Jäger 1 ohne Korrektur. Die postoperative subjektive Refraktion war rechts −0,75/−0,50/A 8° und links −0,75/0,00/A 0°. Die Patientin benötigte damit weder eine Fern- noch eine Lesebrille. Die bestehenden Hornhautnaben waren nicht visusrelevant. Der postoperative Befund ist in Abb. 3 in Mydriasis dargestellt.

Bei Patientin 2 wurde in beiden Augen eine IC-8 (AcuFocus Inc, Irvine, CA, USA) IOL implantiert (+25,0 dpt links und +24,5 dpt rechts), die Zielrefraktion war −0,50 dpt beidseits. Der unkorrigierte postoperative Fernvisus nach 6 Monaten betrug 0,2 LogMAR beidseits monokular und 0,1 LogMAR binokular. Eine Nd:YAG-Laser-Kapsulotomie wurde 7 Monate nach der Kataraktoperation rechts vor links durchgeführt. Danach stabilisierte sich der unkorrigierte Fernvisus bei 0,2 LogMAR bilateral. Mit Minusgläsern (subjektive Refraktion rechts −1,00/−1,50/A 120° und links −1,00/−1,50/A 135°) war der Fernvisus 0,0 LogMAR bilateral. Der unkorrigierte binokulare Nahvisus erreichte Jäger 1.

## Diskussion

Die IC-8 (AcuFocus Inc, Irvine, California, USA) IOL ist eine einteilige hydrophobe Hinterkammer-IOL aus Acryl, in die eine kreisförmige Maske mit einem kleinen zentralen Loch von 1,36 mm eingebracht wurde. Mit der IOL-Maske wird die Tiefenschärfe erweitert. Das IOL-Design basiert auf dem des KAMRA-Hornhaut-Inlays (AcuFocus, Irvine, CA, USA) (korneales Lochblendenkonzept), beruhend auf dem Prinzip der Optik mit „small-aperture“ [3]. Das korneale Lochblendenkonzept sowie die IOL mit Lochblende zeigten vergleichbare Schärfentiefebereiche. Beide Konzepte bieten eine größere Schärfentiefe im Vergleich zu einer konventionellen IOL [4]. Dick et al. haben deshalb diese IOL zur Presbyopiekorrektur monokular sowie binokular implantiert. Diese Fallserie zeigte, dass die bilaterale Implantation der IC-8 (AcuFocus Inc, Irvine, CA, USA) IOL zu einem erweiterten Schärfebereich führt mit besserem Nah- und Intermediärvisus als nach monokularer Implantation und ähnlichen potenziellen Komplikationen im Vergleich zu anderen Premium-IOLs. Die Patientenzufriedenheit war jedoch in der unilateralen Gruppe besser [2, 6].

Agarwal et al. haben die IC-8 (AcuFocus Inc, Irvine, CA, USA) IOL monokular in das nichtdominante Auge nach Laser-in-situ-Keratomileusis (LASIK) implantiert mit positiven Ergebnissen für den Fern- und Nahvisus [1]. Die IC-8 (AcuFocus Inc, Irvine, CA, USA) IOL wurde des Weiteren monokular in das nichtdominante Auge bei Patienten mit hohem Astigmatismus nach perforierender Keratoplastik, radiärer Keratotomie oder bei Keratokonus implantiert. Dazu konnten Hoshmand et al. nachweisen, dass ein Auge mit einer IC-8 (AcuFocus Inc, Irvine, CA, USA) IOL einen Astigmatismus von bis zu 1,5 dpt kompensieren kann [6]. Shajari et al. zeigten, dass diese IOL einen hohen Sicherheitsindex und eine hohe Zufriedenheitsrate hat und bei irregulärem Hornhautastigmatismus zu einer besseren Sehqualität führen kann [9].

Unsere Ergebnisse bestätigen eine Verbesserung der Sehschärfe nach Implantation der IC8 (AcuFocus Inc, Irvine, CA, USA) IOL sowohl monokular im nichtdominanten Auge als auch binokular. Beide Patienten waren mit ihrem postoperativen Fern- und Nahvisus sehr zufrieden.

Darüber hinaus ist zu beachten, dass bei Patienten nach RK die Lage der Inzision bei der Kataraktoperation eine entscheidende Rolle spielt. Die Inzision sollte nicht im Bereich der Keratotomienarben liegen, sonst besteht die Gefahr, die sich öffnenden Keratotomienarben am Ende der Operation nicht mehr dicht zu bekommen.

Es wird vermutet, dass sich nach der Implantation einer IC-8 (AcuFocus Inc, Irvine, CA, USA) IOL rasch eine Hinterkapselfibrose entwickelt. Eine Nd:YAG-Kapsulotomie kann von einem erfahrenen Operateur unter Umgehung der Maske nach dem Omegamuster durchgeführt werden [7].

Die IC-8 (AcuFocus Inc, Irvine, CA, USA) IOL kann entweder bilateral oder nur in das nichtdominante Auge bei Patienten nach keratorefraktivem Eingriff implantiert werden. Sie stellt eine sinnvolle Option zur Korrektur des irregulären Astigmatismus dar.

## Fazit für die Praxis

- Die IC-8 (AcuFocus Inc, Irvine, CA, USA) IOL erlaubt eine Wiederherstellung der Nahvisus und des Sehens in mittlerer Distanz durch Erzeugung einer größeren Tiefenschärfe.
- Sie kann entweder binokular oder monokular in das nichtdominante Auge implantiert werden.
- Die IOL-Berechnung ist eine Herausforderung und benötigt die Kombination von „totaler zentraler cornealer Brechkraft“ mittels Pentacam (Oculus Optikgeräte GmbH, Wetzlar, Deutschland) und optischer Biometrie (Haigis-Formel).
- Die IC-8 (AcuFocus Inc, Irvine, CA, USA) IOL stellt eine sehr gute Behandlungsoption bei irregulärem Astigmatismus z. B. nach keratorefraktivem Eingriff dar.

## References

[1] Agarwal S, Thornell EM (2018). Cataract surgery with small-aperture intraocular lens after previous corneal refractive surgery: visual outcomes and spectacle independence. J Cataract Refract Surg.

[2] Dick HB, Elling M, Schultz T (2018). Binocular and monocular implantation of small-aperture Intraocular lenses in cataract surgery. J Refract Surg.

[3] El-Husseiny M, Daas L, Viestenz A, Langebucher A, Seitz B (2017). Das KAMRA-Inlay. Ein realistischer Ansatz ?. Ophthalmologe.

[4] Eppig T, Spira C, Seitz B, Szentmáry N, Langenbucher A (2016). A comparison of small aperture implants providing increases depth of focus in pseudophakic eyes. Z Med Phys.

[5] Grabner G, Ang RE, Vilupuru S (2015). The small-aperture IC-8 Intraocular lens: a new concept for added depth of focus in cataract patients. Am J Ophthalmol.

[6] Hooshmand J, Allen P, Huynd T, Cha C, Singh R, Moshegov C, Agarwal S, Thornell E, Vote BJ (2019). Small aperture IC-8 intraocular lens in cataract patients: achieving extended depth of focus through small aperture optics. Eye (Lond).

[7] Schojai M, Schultz T, Jerke C, Böcker J, Dick HB (2010). Visual performance comparison of 2 extended depth-of-focus intraocular lenses. J Cataract Refract Surg.

[8] Seitz B, Langebucher A (2000). Intraocular lens power calculation in eyes after corneal refractive surgery. J Refract Surg.

[9] Shajari M, Mackert MJ, Langer J, Kreutzer T, Wolf A, Kohnen T, Priglinger S, Mayer WJ (2010). Safety and efficacy of a small-aperture capsular bag-fixated intraocular lens in eyes with severe coreal irregularities. J Cataract Refract Surg.

